# The complete mitochondrial genome of the rodent flea *Nosopsyllus laeviceps*: genome description, comparative analysis, and phylogenetic implications

**DOI:** 10.1186/s13071-024-06329-y

**Published:** 2024-06-11

**Authors:** Yi-Tian Fu, Ying Xun, Yan-Yan Peng, Yu Zhang, Xiang Wu

**Affiliations:** 1 Department of Parasitology, Xiangya School of Basic Medicine, Central South University, Changsha, 410013 Hunan China; 2 Research Center for Parasites & Vectors, College of Veterinary Medicine, Hunan Agricultural University, Changsha, 410128 Hunan China

**Keywords:** Flea, *Nosopsyllus laeviceps*, Mitochondrial genome, Comparative mitogenomics, Phylogenetics

## Abstract

**Background:**

Fleas are one of the most common and pervasive ectoparasites worldwide, comprising at least 2500 valid species. They are vectors of several disease-causing agents, such as *Yersinia pestis*. Despite their significance, however, the molecular genetics, biology, and phylogenetics of fleas remain poorly understood.

**Methods:**

We sequenced, assembled, and annotated the complete mitochondrial (mt) genome of the rodent flea *Nosopsyllus laeviceps* using next-generation sequencing technology. Then we combined the new mitogenome generated here with mt genomic data available for 23 other flea species to perform comparative mitogenomics, nucleotide diversity, and evolutionary rate analysis. Subsequently, the phylogenetic relationship within the order Siphonaptera was explored using the Bayesian inference (BI) and maximum likelihood (ML) methods based on concentrated data for 13 mt protein-coding genes.

**Results:**

The complete mt genome of the rodent flea *N. laeviceps* was 16,533 base pairs (bp) in a circular DNA molecule, containing 37 typical genes (13 protein-coding genes, 22 transfer RNA [tRNA] genes, and two ribosomal RNA [rRNA] genes) with one large non-coding region (NCR). Comparative analysis among the order Siphonaptera showed a stable gene order with no gene arrangement, and high AT content (76.71–83.21%) with an apparent negative AT and GC skew except in three fleas *Aviostivalius klossi bispiniformis*, *Leptopsylla segnis*, and *Neopsylla specialis*. Moreover, we found robust evidence that the cytochrome *c* oxidase subunit 1 (*cox1*) gene was the most conserved protein-coding gene (Pi = 0.15, non-synonymous/synonymous [Ka/Ks] ratio = 0.13) of fleas. Phylogenomic analysis conducted using two methods revealed different topologies, but both results strongly indicated that (i) the families Ceratophyllidae and Leptopsyllidae were paraphyletic and were the closest to each other, and (ii) the family Ctenophthalmidae was paraphyletic.

**Conclusions:**

In this study, we obtained a high-quality mt genome of the rodent flea *N. laeviceps* and performed comparative mitogenomics and phylogeny of the order Siphonaptera using the mt database. The results will enrich the mt genome data for fleas, lay a foundation for the phylogenetic analysis of fleas, and promote the evolutionary analysis of Siphonaptera.

**Graphical Abstract:**

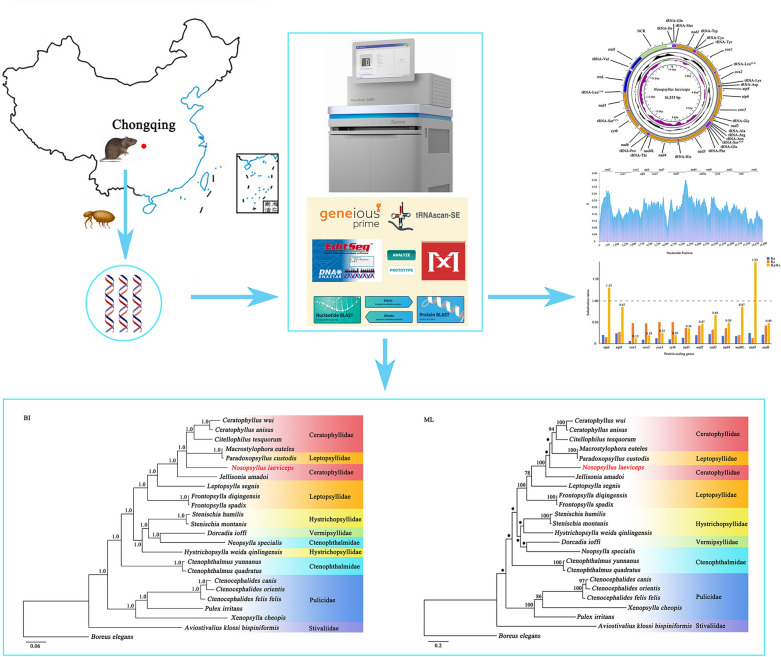

**Supplementary Information:**

The online version contains supplementary material available at 10.1186/s13071-024-06329-y.

## Background

Fleas (Insecta: Siphonaptera) are one of the most common hematophagous ectoparasites of mammals, including humans [1, 2]. They are small, wingless, widespread, and known for their ability to jump [1, 3]. Over 2500 valid species from 16 families and 238 genera have been recognized around the world [1, 4]. Fleas are of great medical and veterinary significance as major vectors of most disease-causing agents to humans and animals worldwide, including *Yersinia pestis* (plague), *Rickettsia typhi* (murine typhus), *Francisella tularensis* (tularemia), and *Bartonella henselae* (cat scratch disease) [5–7]. Additionally, they serve as an intermediate host of pathogenic agents such as *Dipylidium caninum* [8].

Accurate identification and taxonomy of flea species are essential, with important implications for studying their molecular genetics, biology, and phylogenetics. However, the traditional morphological methods are often not efficient in identifying species that are cryptic, morphologically similar, or inconspicuous, which results in some incorrect classifications and phylogenetic relationships [9]. The newly developing molecular methods, particularly mitochondrial (mt) genomics, have been proven more accurate and convenient for flea systematics and phylogenetics [10–12]. It is now generally accepted that the order Siphonaptera is monophyletic using mt genomic data [10, 12, 13], but many extant families among the order Siphonaptera are presented as paraphyletic and controversial [12, 14, 15]. Nevertheless, the available molecular information on fleas is still scarce: only 23 flea species have been sequenced and deposited in GenBank to date. This lack of knowledge of mt genomics constitutes a major limitation for systematic and phylogenetic studies of fleas. Hence, there is a need for further decoding of the mt genomes of flea species.

The family Ceratophyllidae, one of the most common flea families, consists of rodent and avian fleas that predominantly associate with sciurids and specific cricetids [14]. This family comprises 47 genera, including *Ceratophyllus*, *Jellisonia*, and *Nosopsyllus*. The genus *Nosopsyllus* (Siphonaptera: Ceratophyllidae), also regarded as *Ceratophyllus* or *Gerbillophillus*, is native to the Palearctic regions and contains more than 60 recognized species [16], which mostly parasitize rodents but occasionally parasitize domestic mammals. Some of them can bite humans and are critical vectors of pathogenic agents causing enzootic plague among Palearctic regions [17, 18]. The rodent flea *Nosopsyllus laeviceps* (Wagner, 1909) comprises three subspecies, *N. laeviceps laeviceps*, *N. l. ellobii*, and *N. l. kuzenkovi*. So far, no whole-mt genomes are available for this large genus. Therefore, the aims of this study were (i) to decode and characterize the complete mt genome of the rodent flea *N. laeviceps*, (ii) to compare and analyze the mitogenome of the rodent flea *N. laeviceps* with other fleas, and (iii) to explore the interordinal phylogenetic relationship of families among the order Siphonaptera using mitogenome information.

## Methods

### Sample collection, observation, washing, and DNA extraction

All procedures involving animals in the present study were approved by the Animal Ethics Committee (no. 201703386). Adults of the rodent flea samples were collected from the body surface of sewer rats *Rattus norvegicus* in Chongqing Municipality, China. All flea specimens were placed into centrifuge tubes after initial washing. Morphological identification of fleas was performed to the genus level preliminarily using a stereoscopic microscope (Nikon SMZ18, Tokyo, Japan) [19]. Then each flea was put into a sterile centrifuge tube with physiological saline solution, followed by oscillation and washing to remove the impurities and dust attached to the body surface. Notably, when a distinct red band was observed in the abdomen of the flea, the abdomen was cut open and the blood cleaned to ensure the accuracy of subsequent DNA extraction. After the above process, the samples were stored separately in 100% ethanol at −40 °C for subsequent molecular study.

Total genomic DNA was extracted from individual fleas using the QIAamp^®^ DNA Micro Kit (QIAGEN, Hilden, Germany) following the manufacturer's instructions. DNA quantity and quality were determined and analyzed using a Qubit 4.0 Fluorometer (Invitrogen, Carlsbad, CA, USA) and 1.0% agarose gel electrophoresis, respectively. Sequence amplification was performed by polymerase chain reaction (PCR)-based sequencing of the mt cytochrome *c* oxidase subunit 1 (*cox1*) and *cox2* genes as described previously [20], and the PCR products were sequenced from both directions (forward and reverse) by Sangon Biotech Company (Shanghai). Molecular identification was further completed by nucleotide sequence alignment with fleas deposited in the GenBank database.

### Mitochondrial genome sequencing, assembly, annotation, and visualization

A genomic DNA library (350-base-pair [bp] inserts) was constructed using the Illumina NovaSeq 6000 platform (Illumina, San Diego, CA, USA). The raw reads were produced in FASTQ format through the paired-end 250 (PE250) sequencing strategy and then filtered by removing adaptor reads, highly repetitive reads, “N”-rich reads, and low-quality reads using Fastp v.0.19.7 software [21]. The whole mt genome was assembled using the Map to Reference tool in Geneious Prime (https://www.geneious.com) with the amplified *cox1* and *cox2* gene sequences as the initial references. The assembly criteria were a minimum overlap identity of 99% and minimum overlap of 150 bp. The assembly was considered complete when it generated a large contig ending with overlapping fragments.

Thirteen protein-coding genes were predicted and annotated using ORFfinder (https://www.ncbi.nlm.nih.gov/orffinder/) and Basic Local Alignment Search Tool (BLAST) searches of the NCBI database, and 22 transfer RNA (tRNA) genes and the corresponding secondary structures were recognized by ARWEN [22] and tRNAscan-SE [23]. Two ribosomal RNA (rRNA) genes were determined by alignment and comparison with other available mt genomes of fleas. All genes were checked manually in the MITOS WebServer (http://mitos.bioinf.uni-leipzig.de/index.py) [24]. Sequence alignment was analyzed using MEGA 11 software [25]. The mt genome of the rodent flea *N. laeviceps* was visualized using the Proksee system (https://proksee.ca/).

### Sequence analysis

The base content was computed using DNASTAR v.5.0, and GC and AT skews were then calculated with the following formulas: GC skew = (G − C)/(G + C), AT skew = (A − T)/(A + T). The complete mt genomes among the rodent fleas generated in this study were compared with those of fleas available in the GenBank database with respect to length, gene order, and AT content. DnaSP v.6 software [26] was used to perform the nucleotide diversity and evolutionary rate analysis. The former was calculated through a sliding window with the parameters of window size = 300 and step size = 25; and the latter was analyzed by the non-synonymous (Ka)/synonymous (Ks) substitutions ratios.

### Phylogenetic analysis

A total of 23 available flea species, along with the outgroup of the scorpion fly *Boreus elegans* (GenBank accession number: HQ696579), were selected for phylogenetic analysis (Table S1). Amino acid sequences of 13 mt protein-coding genes were aligned using MAFFT 7.122 [27]. The aligned sequences were then concatenated to form a single dataset. Ambiguous positions were excluded using Gblocks 0.91b [28] with default parameters.

Phylogenetic analyses were performed through Bayesian inference (BI) and maximum likelihood (ML) methods. For BI analysis, the phylogenetic tree was constructed using MrBayes 3.2.6. [30], and the most suitable model of evolution was selected automatically by this program. Four independent Markov chains were run simultaneously for 1 million metropolis-coupled Markov chain Monte Carlo generations, sampling a tree every 100 generations. The first 2500 trees represented burn-in, and the remaining trees were tested for stability of likelihood values and used to compute Bayesian posterior probability (Bpp). We assumed that stationarity had been reached when the estimated sample size (ESS) was greater than 100, the potential scale reduction factor (PSRF) approached 1.0, and the average standard deviation of split frequencies (ASDSF) was < 0.01.

For the ML method, MtArt + I + G + F was selected as the best model by ProtTest 3.4 [30] based on the Akaike information criterion (AIC). The gamma shape was 0.66 under four rate categories, and the proportion of invariable sites was 0.23. The tree topology search was set from the subtree pruning and regrafting (SPR) method. The phylogenetic tree was then constructed using PhyML 3.1 [31] with a BioNJ starting tree. The bootstrap value was calculated using 100 bootstrap replicates and indicated at nodes. The phylogenetic tree was visualized using FigTree v.1.42.

## Results

### Identification of the rodent flea *N. laeviceps*

All specimens collected in this study showed typical generic morphological features of *Nosopsyllus* spp.: (1) Their heads are round, with one pair of well-developed eyes and antennas. (2) The anterior pectoral segment has spines, and the dorsal spines are about the same length as the backplane. The front femur has a lateral setae row. (3) Metanotum and abdominal tergites present a setae row; the anterior one is usually vestigial or absent. (4) The eighth dorsal plate has no spines.

We obtained an abundance of *cox1* and *cox2* gene sequences from the flea samples. BLAST results showed that the partial *cox1* gene of specimens had 96.6% and 96.4% similarity to the subspecies *N. l. ellobii* (GenBank accession number: KM890985) and *N. l. kuzenkovi* (GenBank accession number: KM890987), respectively. The partial sequences of the *cox2* gene of specimens had 98.1%, 98.1%, and 97.6% similarity to the rodent flea *N. l. laeviceps* (GenBank accession number: MF045767), *N. l. ellobii* (GenBank accession number: KM890852), and *N. l. kuzenkovi* (GenBank accession number: KM890858), respectively.

### Characterization of the mt genome of the rodent flea *N. laeviceps*

**Fig. 1:**
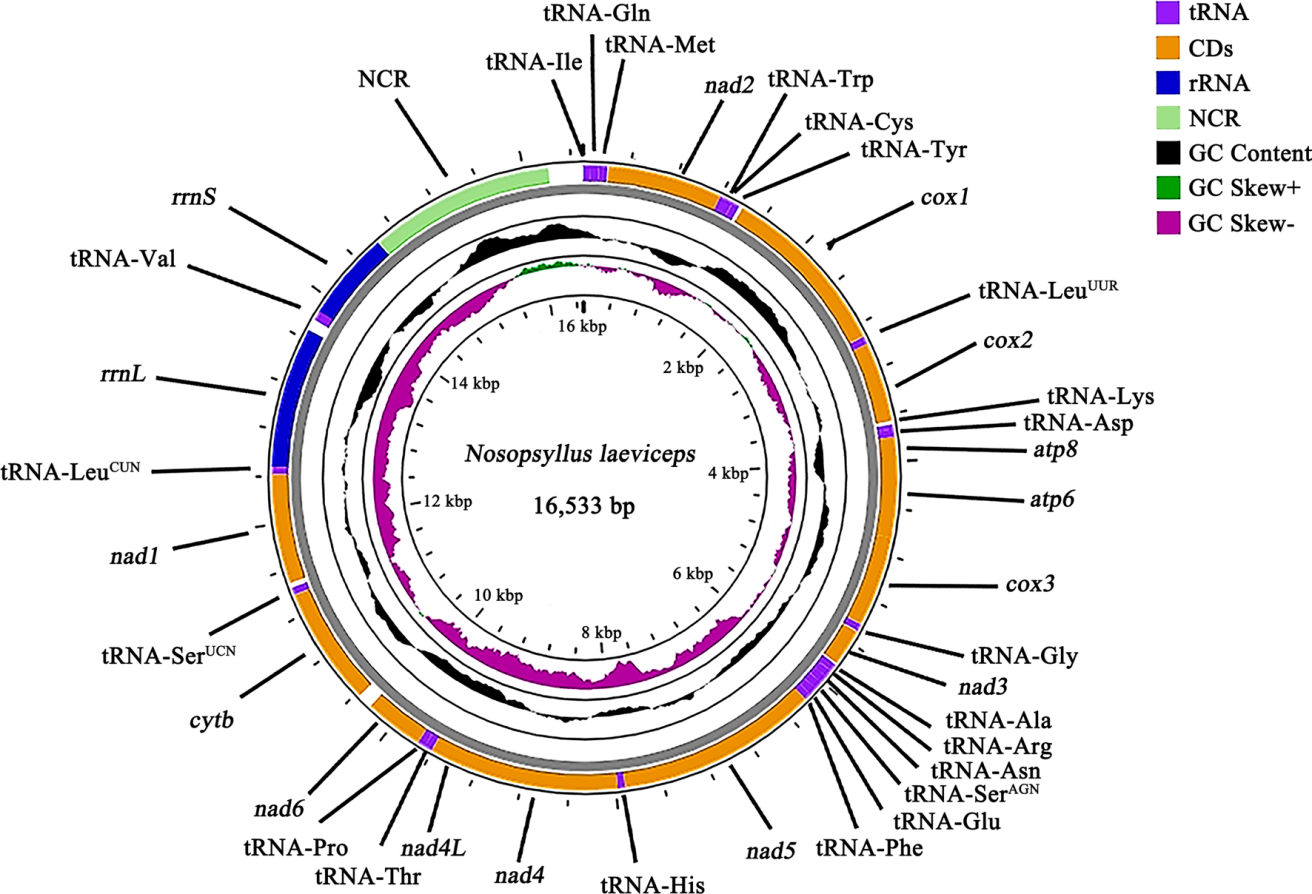
The complete mitochondrial genome of rodent flea *Nosopsyllus laeviceps*. Gene scaling is only approximate

There were over 3 GB of Illumina short-read sequence datasets produced from the DNA library of the rodent flea *N. laeviceps***,** including 10,698,472 × 2 clean reads. The complete mt genome presenting as a typical circular structure was 16,533 bp in size (Fig. 1), and has been deposited in the NCBI database (GenBank accession no. PP838812). The mt genomes recognized 37 typical genes of metazoan animals, containing 13 protein-coding genes (adenosine triphosphate [ATP] synthase F0 subunit 6 [*atp6*], *atp8*, cytochrome *c* oxidase subunits 1–3 [*cox1–3*], cytochrome *b* [*cytb*], nicotinamide adenine dinucleotide [NADH] dehydrogenase subunits 1–6 [*nad1–6*], and *nad*4L), two rRNA genes (large subunit rRNA and small subunit rRNA), and 22 tRNA genes (Table 1, Fig. 1). A total of 23 genes were on the heavy strand, while the other 14 genes were on the light strand (Table 1). There were 13 gene-overlapped locations with 1–19 bp per location. Likewise, the intergenic regions were discovered in 14 different locations, with the longest located between the large subunit of rRNA (*rrnL*) and tRNA-Val (valine) genes (99 bp) (Table 1).

**Table 1:** Organization of the mitochondrial genomes of the rodent flea *Nosopsyllus laeviceps*

Gene/region	Positions	Strand	Size (bp)	Number of aa^a^	Ini/Ter codons^b^	Anticodon	In^c^
tRNA-Ile (I)	1–64	H	64			GAT	0
tRNA-Gln (Q)	133–65	L	69			TTG	−1
tRNA-Met (M)	133–206	H	74			CAT	+5
*nad2*	212–1211	H	1000	333	ATA/T		0
tRNA-Trp (W)	1212–1276	H	65			TCA	−8
tRNA-Cys (C)	1329–1269	L	61			GCA	0
tRNA-Tyr (Y)	1393–1330	L	64			GTA	0
*cox1*	1394–2926	H	1533	510	GTG/TAA		+4
tRNA-Leu^UUR^ (L_2_)	2931–2994	H	64			TAA	+1
*cox2*	2996–3676	H	681	226	ATG/TAA		+32
tRNA-Lys (K)	3680–3748	H	70			CTT	−1
tRNA-Asp (D)	3748–3812	H	65			GTC	+1
*atp8*	3814–3996	H	183	60	ATG/TAA		−19
*atp6*	3978–4664	H	687	228	ATT/TAA		−1
*cox3*	4664–5435	H	772	257	ATG/T		+4
tRNA-Gly (G)	5440–5500	H	61			TCC	−2
*nad3*	5499–5838	H	340	113	ATT/T		0
tRNA-Ala (A)	5839–5902	H	64			TGC	+1
tRNA-Arg (R)	5904–5967	H	64			TCG	−4
tRNA-Asn (N)	5964–6031	H	68			GTT	−2
tRNA-Ser^AGN^ (S_1_)	6030–6099	H	70			TCT	−2
tRNA-Glu (E)	6098–6162	H	65			TTC	−3
tRNA-Phe (F)	6219–6160	L	60			GAA	0
*nad5*	7938–6220	L	1719	572	ATG/TAG		+1
tRNA-His (H)	8001–7940	L	62			GTG	0
*nad4*	9337–8002	L	1336	445	ATG/T		−7
*nad4L*	9624–9331	L	294	97	ATG/TAA		+2
tRNA-Thr (T)	9627–9692	H	66			TGT	−1
tRNA-Pro (P)	9756–9692	L	65			TGG	+1
*nad6*	9758–10,273	H	516	171	ATT/TAA		−1
*cytb*	10,273–11,412	H	1140	379	ATG/TAA		+2
tRNA-Ser^UCN^ (S_2_)	11,415–11,477	H	63			TGA	+41
*nad1*	12,451–11,519	L	933	310	ATG/TAA		+1
tRNA-Leu^CUN^ (L_1_)	12,514–12,453	L	62			TAG	0
*rrnL*	13,710–12,515	L	1196				+99
tRNA-Val (V)	13,877–13,810	L	68			TAC	0
*rrnS*)	14,651–13,878	L	774				0
AT-loop reg*ion*	14,652–16,533		1882				0

aa, amino acid

^a^ The inferred length of the aa sequence of 13 protein-coding genes

^b^ Ini/Ter codons: initiation and termination codons

Almost all protein-coding genes in the mt genome of the rodent flea *N. laeviceps* used the common start codons, including ATT (*atp6*, *nad3*, and *nad6*), ATG (*atp8*, *cox2*, *cox3*, *cytb*, *nad1*, *nad4*, *nad4L*, and *nad5*), and ATA (*nad2*), while the *cox1* gene used GTG as the start codon (Table 1). Complete stop codon TAA was the most frequently used, followed by the incomplete stop codon T. Nevertheless, the usual stop codon TAG was used only once in the *nad5* gene (Table 1). Furthermore, there was only one non-coding region (NCR) in the mt genome of *N. laeviceps* (Table 1, Fig. 1). The large NCR, also referred to as the control region, involving the regulation of DNA replication was located between the tRNA-Ile (I) gene and *rrnS* gene, and was 1882 bp in size. The AT content was 71.3%. Compared with other available mt genomes of fleas, there are only three Pulicidae species containing two NCRs. The cat flea *Ctenocephalides felis felis* and the human flea *Pulex irritans* had two long NCRs, while the dog flea *C. canis* had two short NCRs.

### Comparative mt genomics analysis among the order Siphonaptera

A comprehensive comparison of the sizes, AT content, and base skews of the nucleotide sequences from Siphonaptera species is given in Table 2. About half of the mt genomes of fleas were approximately 15,000 bp in length, while three Pulicidae fleas *Ctenocephalides felis felis*, *Ctenocephalides orientis*, and *Pulex irritans* showed an unusual size of over 20,000 bp. The rodent flea *N. laeviceps* had high AT content (78.10%) and obviously exhibited negative AT skew (−2.87) and GC skew (−16.53) (Table 2). It is notable that AT skew and GC skew were all negative among the order Siphonaptera except for three fleas *Leptopsylla segnis*, *Aviostivalius klossi bispiniformis*, and *Neopsylla specialis*, with the first conversely exhibiting a positive AT skew (2.36) and GC skew (24.77), while the latter two both displayed a neutral AT skew (0) and negative GC skew (−16.99 and −25.12) (Table 2). Compared with other mt genomes of fleas, we found that the mt gene order of the rodent flea *N. laeviceps* was identical to that of other fleas. The gene order was stable, with no gene arrangement among the order Siphonaptera.

**Table 2:** Comparison of the mt genomes from Siphonaptera species, including the rodent flea *Nosopsyllus laeviceps*

Family	Species	Size (bp)	A + T (%)	AT skew	GC skew
Ceratophyllidae	*Ceratophyllus anisus*	15,875	78.54	−2.20	−23.11
	*Ceratophyllus wui*	18,081	76.71	−1.66	−18.33
	*Citellophilus tesquorum*	15,345	78.1	−2.92	−21.75
	*Jellisonia amadoi*	17.031	79.17	−2.03	−25.97
	*Macrostylophora euteles*	16,027	77.59	−0.79	−26.82
	***Nosopsyllus laeviceps***	**16,533**	**78.10**	−**2.87**	−**16.53**
Ctenophthalmidae	*Ctenophthalmus quadratus*	15,938	79.45	−1.35	−22.53
	*Ctenophthalmus yunnanus*	15,801	79.36	−1.59	−22.77
	*Neopsylla specialis*	16,820	77.27	0.01	−25.12
Hystrichopsyllidae	*Hystrichopsylla weida qinlingensis*	17,173	80.59	−2.97	−22.10
	*Stenischia humilis*	15,617	78.00	−1.10	−23.82
	*Stenischia montanis*	15,651	77.29	−1.16	−23.73
Leptopsyllidae	*Frontopsylla diqingensis*	15,878	79.33	−3.50	−21.43
	*Frontopsylla spadix*	15,085	78.83	−3.62	−21.46
	*Leptopsylla segnis*	15,785	78.89	2.36	24.77
	*Paradoxopsyllus custodis*	15,375	76.79	−0.77	−25.89
Pulicidae	*Ctenocephalides canis*	15,609	78.52	−1.71	−18.16
	*Ctenocephalides felis felis*	20,911	82.88	−4.42	−23.71
	*Ctenocephalides orientis*	22,189	83.21	−5.11	−25.79
	*Pulex irritans*	20,337	80.02	−2.71	−14.61
	*Xenopsylla cheopis*	18,902	82.83	−1.05	−22.12
Stivaliidae	*Aviostivalius klossi bispiniformis*	16,593	79.04	0	−16.99
Vermipsyllidae	*Dorcadia ioffi*	16,785	80.71	−0.63	−19.75

The species in bold is the rodent flea collected in this study

### Sliding window analysis and non-synonymous/synonymous substitution ratio

**Fig. 2:**
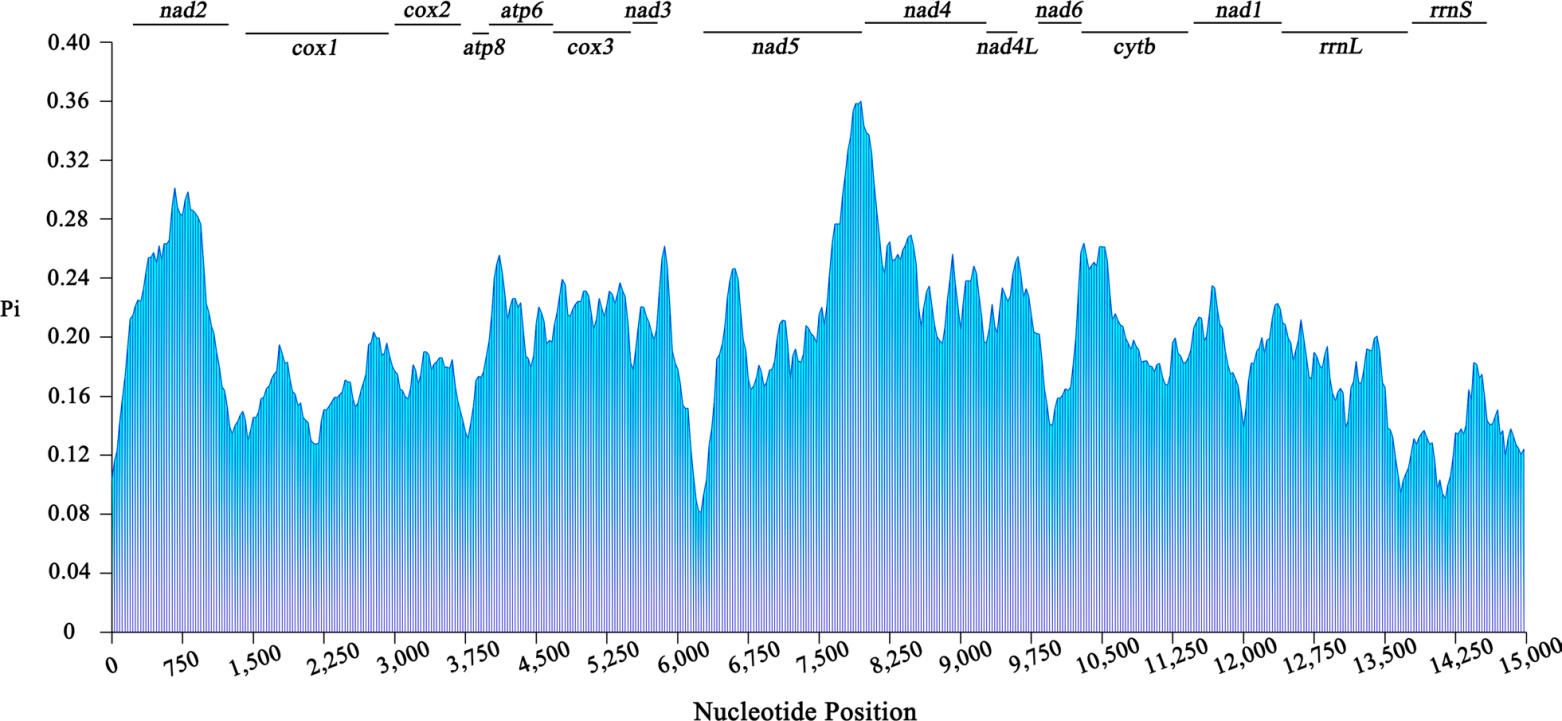
Sliding window analysis of the alignment of complete mitochondrial genomes except for non-coding regions of Siphonaptera insects. The average nucleotide diversity value of each gene is indicated above the graph

**Fig. 3:**
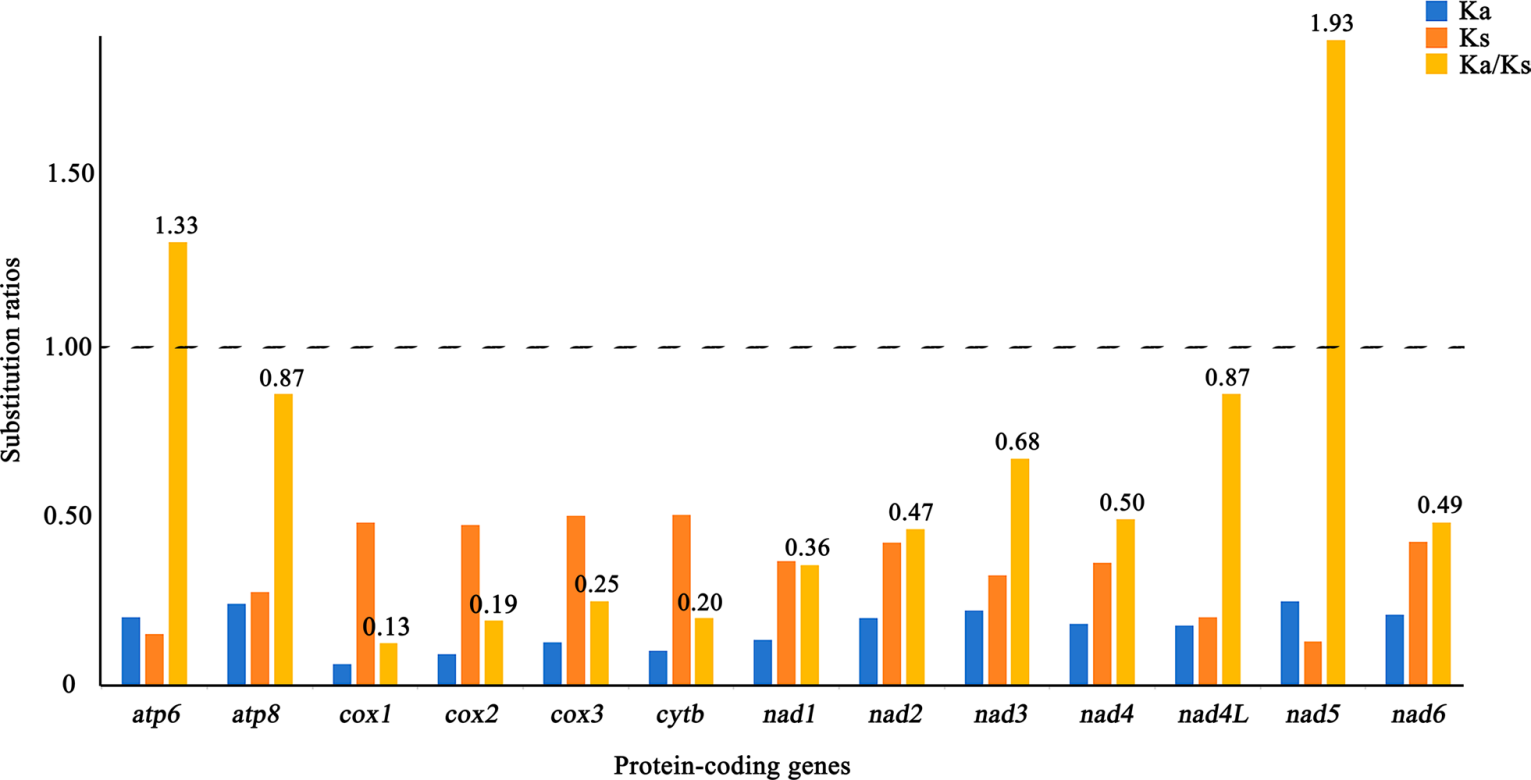
Substitution ratios in the mitochondrial genomes of fleas. The rate of non-synonymous (Ka) and synonymous (Ks) substitutions, and the expected ratios (Ka/Ks) for individual protein-coding genes are shown

High nucleotide sequence variability was observed in the *nad2* and *nad5* genes (peak Pi > 0.30), while low sequence variability was found in the *rrnS*, *rrnL*, and *cox1* genes (low value < 0.13) (Fig. 2). Likewise, the Ka/Ks substitution ratio of 13 protein-coding genes showed that the *nad5* gene appeared to have the highest Ka/Ks ratio (1.93), while the *cox1* gene had the lowest ratio (0.13) (Fig. 3). Here, the Ka/Ks ratios of two protein-coding genes (*atp6* and *nad5*) were significantly higher than 1.00 (Fig. 3), indicating that these mt genes of fleas have evolved under positive selective pressure with a high evolutionary rate. The other genes with Ka/Ks ratios lower than 1.00 were under purified selection.

### Phylogenetic relationships

**Fig. 4:**
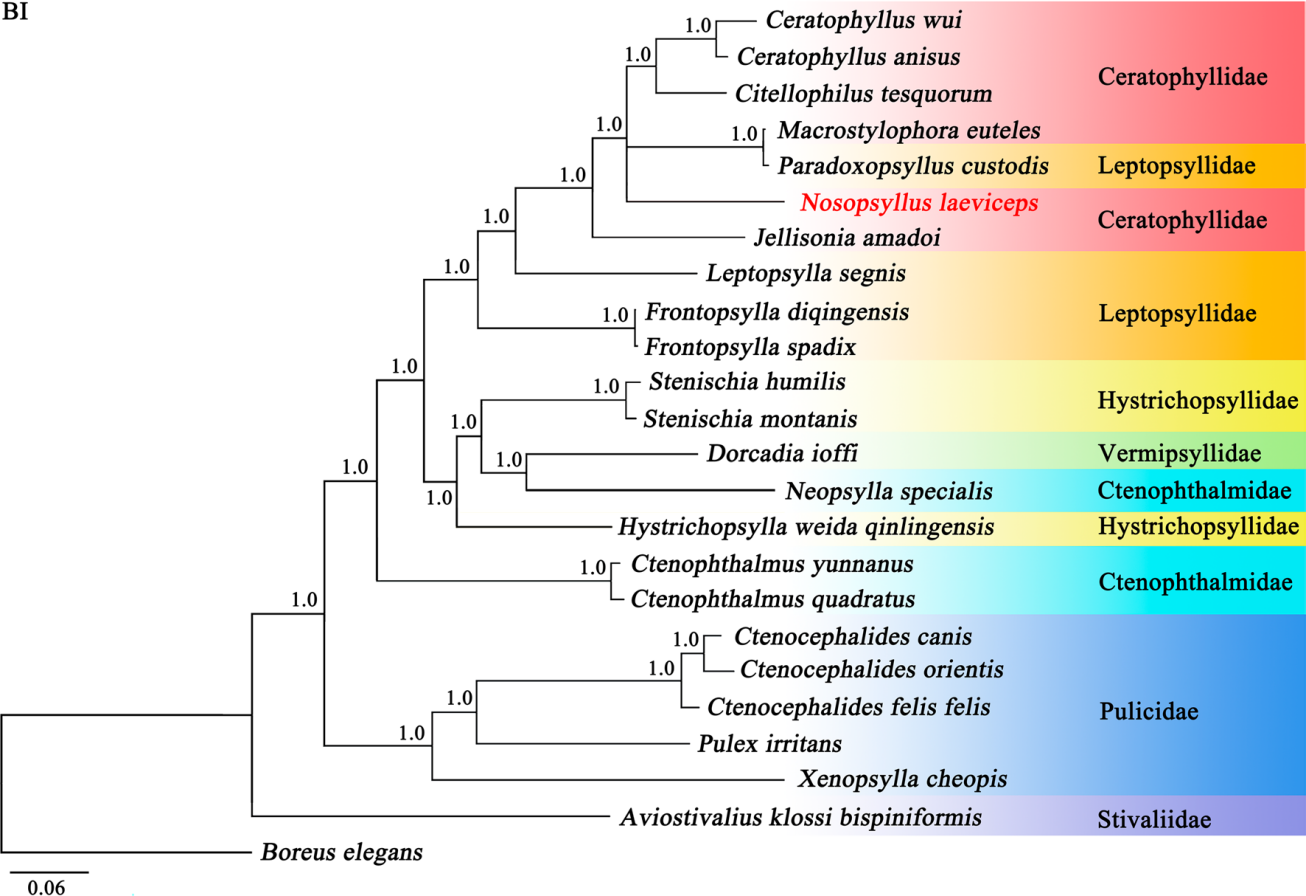
Phylogenetic relationships among 24 species of Siphonaptera insects inferred from Bayesian inference (BI) analysis of deduced amino acid sequences of 13 mitochondrial proteins. *Boreus elegans* (GenBank accession number HQ696579) was used as the outgroup. The rodent flea *Nosopsyllus laeviceps* in the present study is shown in red font. Bayesian posterior probabilities (Bpp) are indicated at nodes

**Fig. 5:**
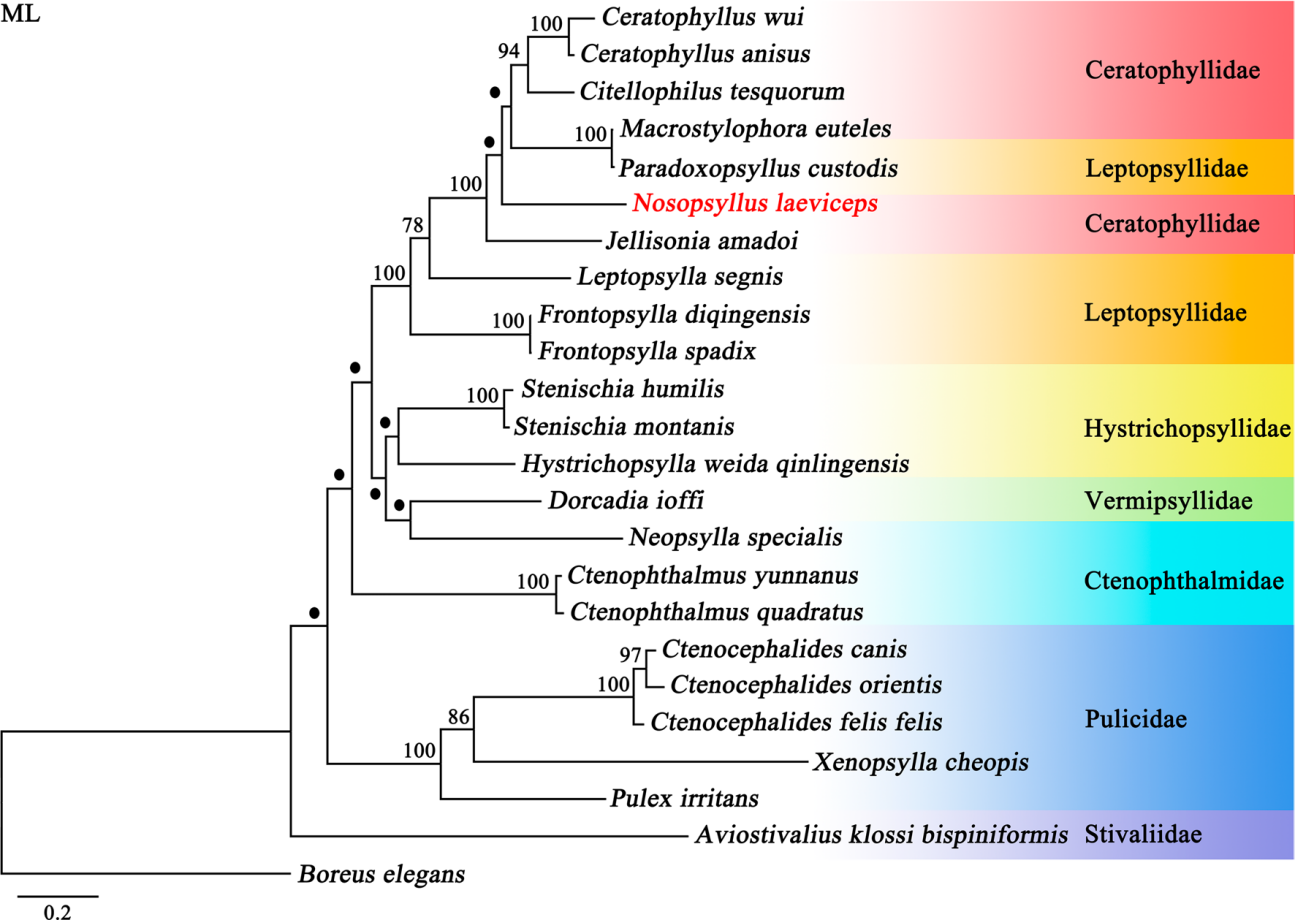
Phylogenetic relationships among 24 species of Siphonaptera insects inferred from maximum likelihood (ML) analysis of deduced amino acid sequences of 13 mitochondrial proteins. *Boreus elegans* (GenBank accession number HQ696579) was used as the outgroup. The rodent flea *Nosopsyllus laeviceps* in the present study was shown in red font. Bootstrap values (Bv) were indicated at nodes. Nodes < 70 are shown in a solid black circle

Phylogenetic analysis using the BI and ML methods based on 13 protein-coding genes of 24 flea species showed different topologies (Figs. 4 and 5). Two topologies indicated that *N. laeviceps* was more closely related to ([*Paradoxopsyllus custodis* + *Macrostylophora euteles*] + [*Ceratophyllus anisus* + *Ceratophyllus wui* + *Citellophilus tesquorum*]), with strong BI support (Bpp = 1.0) and weak ML support (Bv < 70) (Figs. 4 and 5), indicating that the family Ceratophyllidae was paraphyletic. Three other members within the family Leptopsyllidae (*Frontopsylla spadix*, *Frontopsylla diqingensis*, and *Leptopsylla segnis*) were not grouped together with high statistical value (Bpp = 1.0; Bv = 100) (Figs. 4 and 5), indicating that the family Leptopsyllidae was paraphyletic. All flea species included in the present study were clustered in a large clade, with the exception of *Aviostivalius klossi bispiniformis* (Siphonaptera: Stivaliidae) (Bpp = 1.0; Bv < 70), which was in the outermost clade (Figs. 4 and 5), and the family Pulicidae formed a monophyletic group (Bpp = 1.0; Bv = 100). In BI topology, the families Hystrichopsyllidae and Ctenophthalmidae were paraphyletic, as the fleas *Hystrichopsylla weida qinlingensis*, *Stenischia humilis*, and *Stenischia montanis* were clustered separately with the family Vermipsyllidae (Fig. 4). However, ML analysis supported the monophyly of the family Hystrichopsyllidae but rejected the monophyly of the family Ctenophthalmidae with a low node.

## Discussion

Fleas are one of the most common arthropod-borne organisms, with global distribution [6]. In the present study, we characterized and analyzed the complete mt genome of *N. laeviceps* for the first time, and then performed comparative mitogenomics, nucleotide diversity, evolutionary rate analysis, and phylogenetic analysis using an mt database. The Ka/Ks substitution ratios of mt protein-coding genes represent the molecular evolutionary rates in one taxon of closely related species [32]. They reflect the kind of selective pressures the mt genes undergo during their evolution. When the Ka/Ks ratio was equal to 1, it indicated that the mt gene was under neutral selective pressure, where harmful mutations and beneficial mutations counterbalance each other. When the Ka/Ks ratio was greater than 1, it meant that harmful mutations accumulated in the reciprocal evolutionary process of mt genes and nuclear genes. To eliminate harmful mutations, positive selection pressure acts on mt genes, resulting in their adaptive evolution. In contrast, when Ka was smaller than Ks, mt genes are governed by negative selection or purified selection, preventing the amino acid sequence from being altered [33, 34]. The Ka/Ks ratio is usually relevant to sequence conservation, and the results in this study revealed that the *nad5* gene sequence may display greater variation among the order Siphonaptera, while the *cox1* gene may be the most conserved. Nucleotide diversity analysis demonstrated that *rrnL*, *rrnS*, and *cox1* genes were the most highly conserved genes, indicating that they are suitable molecular markers for species identification, while *nad2* and *nad5* genes with a high Pi are more appropriate for the study of species evolution.

The taxonomic status and phylogenetic relationships of Siphonaptera insects have been one of the most stimulating problems in insect taxonomy, systematics, and evolutionary biology For many years, the systematic classification of fleas was based mainly on traditional morphological and physiological characteristics, which has great limitations and a long history of controversy. The development of molecular systematics has provided new insights into the taxonomy and systematics of fleas. Due to the practicability of mt genomic datasets, high phylogenetic signal, and strong statistical support in trees, reanalysis of the phylogenetic relationships using expanded mt datasets is advisable.

Whiting et al. [14] constructed the first comprehensive phylogenetic tree for fleas using concentrated data from four loci: 18S ribosomal DNA (rDNA), 28S rDNA, *cox2*, and elongation factor 1 alpha (EF-1α) genes. To gain a comprehensive understanding of the phylogenetic relationship of fleas, they used 128 different taxa representing 16 families and 83 flea genera with eight outgroups collected globally. Based on the molecular analysis, they proposed that 10 families are monophyletic, including Tungidae, Lycopsyllidae, Pygiopsyllidae, Stivaliidae, Stephanocircidae, Rhopalopsyllidae, Chimaeropsyllidae, Pulicidae, Ischnopsyllidae, and Ceratophyllidae, while the families Hystrichopsyllidae, Ctenophthalmidae, and Leptopsyllidae are paraphyletic. Nevertheless, the results were produced by a few single genes, comprising limited molecular information; thus, they noted that it was essential to clarify the systematics and phylogenetics of fleas using new molecular and morphological data. Previous studies [10, 12, 35] have reconstructed the phylogenetic relationships of fleas using the complete mt genomes, but the fleas included represented no more than 15 species, and therefore did not reflect the whole phylogenetic relationship of fleas. Our phylogenetic analysis results supported the monophyly of Pulicidae but rejected the monophyly of Ctenophthalmidae, Leptopsyllidae, and Ceratophyllidae, which was consistent with a large proportion of previous studies [1, 15, 35–37], but contradicted the results obtained from Whiting et al. [14] using the concentrated four loci [14].

Phylogenetic relationships of fleas are still controversial, despite the extensive studies on flea taxonomy and biology. Mitochondrial genomes contain abundant molecular information that has been widely used for systematics, phylogenetics, population genetics, and evolutionary studies of metazoans over the past three decades [38–42]. It has been shown that mt genomes are suitable molecular tools in species identification, phylogenetic analysis, molecular epidemiology, and other areas of research on fleas [1, 10, 14, 35, 37]. Meanwhile, morphological characteristics and host information remain important information sources for the classification and identification of fleas.

## Conclusions

In this study, we obtained a high-quality mt genome of the rodent flea *N. laeviceps*. Our findings showed that the *cox1* gene is a suitable molecular marker for the identification of fleas. Phylogenetic analysis showed that the families Ceratophyllidae, Ctenophthalmidae, and Leptopsyllidae were paraphyletic and supported the monopoly of the family Pulicidae. Compared with other studies, our phylogeny generated from mt genome datasets showed a different topology. Therefore, more mt genome data would be necessary to resolve the phylogeny of fleas. Our results will enrich the mt genomic data for fleas, lay a foundation for the phylogenetic analyses of fleas, and promote the evolutionary analysis of Siphonaptera species.

### Supplementary Information

Supplementary Material 1.

## Data Availability

The mitochondrial genome sequence of *Nosopsyllus laeviceps* has been deposited in the GenBank database under the accession number PP838812.

## Abbreviations

Mt: Mitochondrial
rRNA: Ribosomal RNA
tRNA: Transfer RNA
*atp6*: ATP synthase F0 subunit 6
*atp8*: ATP synthase F0 subunit 8
*cox1*: Cytochrome *c* oxidase subunit 1
*cox2*: Cytochrome *c* oxidase subunit 2
*cox3*: Cytochrome *c* oxidase subunit 3
*cytb*: Cytochrome b
*nad1*: NADH dehydrogenase subunit 1
*nad2*: NADH dehydrogenase subunit 2
*nad3*: NADH dehydrogenase subunit 3
*nad4*: NADH dehydrogenase subunit 4
*nad4L*: NADH dehydrogenase subunit 4L
*nad5*: NADH dehydrogenase subunit 5
*nad6*: NADH dehydrogenase subunit 6
*rrnL*: Large subunit of rRNA
*rrnS*: Small subunit of rRNA
rDNA: Ribosomal DNA
BI: Bayesian inference
ML: Maximum likelihood
AIC: Akaike information criterion
NCR: Non-coding region
EF-1α: Elongation factor 1 alpha
