# Supplementary material for: The complete mitochondrial genome of the rodent flea Nosopsyllus laeviceps: genome description, comparative analysis, and phylogenetic implications
Source: Parasit Vectors. 2024 Jun 11;17:253. doi: 10.1186/s13071-024-06329-y (PMC11165799; doi:10.1186/s13071-024-06329-y)
Supplement: Supplementary file 1 — Supplementary Material 1. [file 13071_2024_6329_MOESM1_ESM.docx]

**Table S1** Flea species included in the phylogenetic analyses in this study

| Family | Species | GenBank accession number | |  |
| --- | --- | --- | --- | --- |
| Ceratophyllidae | *Ceratophyllus anisus* | | NC_073017 | |
|  | *Ceratophyllus wui* | | NC_040301 | |
|  | *Citellophilus tesquorum* | | NC_088096 | |
|  | *Jellisonia amadoi* | | NC_022710 | |
|  | *Macrostylophora euteles* | | NC_085274 | |
|  | ***Nosopsyllus laeviceps*** | | **PP838812** | |
| Ctenophthalmidae | *Ctenophthalmus quadratus* | NC_072692 | |  |
|  | *Ctenophthalmus yunnanus* | NC_085277 | |  |
|  | *Neopsylla specialis* | NC_073019 | |  |
| Hystrichopsyllidae | *Hystrichopsylla weida qinlingensis* | NC_042380 | |  |
|  | *Stenischia humilis* | NC_073020 | |  |
|  | *Stenischia montanis* | OR780663 | |  |
| Leptopsyllidae | *Frontopsylla diqingensis* | NC_085276 | |  |
|  | *Frontopsylla spadix* | NC_073018 | |  |
|  | *Leptopsylla segnis* | NC_072691 | |  |
|  | *Paradoxopsyllus custodis* | OQ627398 | |  |
| Pulicidae | *Ctenocephalides canis* | NC_063710 | |  |
|  | *Ctenocephalides felis felis* | MW420044 | |  |
|  | *Ctenocephalides orientis* | NC_073009 | |  |
|  | *Pulex irritans* | NC_063709 | |  |
|  | *Xenopsylla cheopis* | MW310242 | |  |
| Stivaliidae | *Aviostivalius klossi bispiniformis* | OR774970 | |  |
| Vermipsyllidae | *Dorcadia ioffi* | NC_036066 | |  |

Note: The species in bold is the rodent flea collected in this study.
